# Effect of Short- and Long-Range Interactions on Trp Rotamer Populations Determined by Site-Directed Tryptophan Fluorescence of Tear Lipocalin

**DOI:** 10.1371/journal.pone.0078754

**Published:** 2013-10-28

**Authors:** Oktay K. Gasymov, Adil R. Abduragimov, Ben J. Glasgow

**Affiliations:** Departments of Pathology and Laboratory Medicine and Ophthalmology and Jules Stein Eye Institute, University of California Los Angeles, Los Angeles, California, United States of America; University of Pittsburgh School of Medicine, United States of America

## Abstract

In the lipocalin family, the conserved interaction between the main α-helix and the β-strand H is an ideal model to study protein side chain dynamics. Site-directed tryptophan fluorescence (SDTF) has successfully elucidated tryptophan rotamers at positions along the main alpha helical segment of tear lipocalin (TL). The rotamers assigned by fluorescent lifetimes of Trp residues corroborate the restriction expected based on secondary structure. Steric conflict constrains Trp residues to two (*t*, *g* ^−^) of three possible χ_1_ (*t*, *g* ^−^, *g* ^+^) canonical rotamers. In this study, investigation focused on the interplay between rotamers for a single amino acid position, Trp 130 on the α-helix and amino acids Val 113 and Leu 115 on the H strand, i.e. long range interactions. Trp130 was substituted for Phe by point mutation (F130W). Mutations at positions 113 and 115 with combinations of Gly, Ala, Phe residues alter the rotamer distribution of Trp130. Mutations, which do not distort local structure, retain two rotamers (two lifetimes) populated in varying proportions. Replacement of either long range partner with a small amino acid, V113A or L115A, eliminates the dominance of the *t* rotamer. However, a mutation that distorts local structure around Trp130 adds a third fluorescence lifetime component. The results indicate that the energetics of long-range interactions with Trp 130 further tune rotamer populations. Diminished interactions, evident in W130G113A115, result in about a 22% increase of α-helix content. The data support a hierarchic model of protein folding. Initially the secondary structure is formed by short-range interactions. TL has non-native α-helix intermediates at this stage. Then, the long-range interactions produce the native fold, in which TL shows α-helix to β-sheet transitions. The SDTF method is a valuable tool to assess long-range interaction energies through rotamer distribution as well as the characterization of low-populated rotameric states of functionally important excited protein states.

## Introduction

Molecular interactions with proteins are regulated through conformational changes that are hierarchical in time and space [1]–[3]. Conformational changes that determine function have been considered as induced fit [4]–[6], conformational selection [7]–[11], and allosteric effect [12] mechanisms. However, in some cases there are no distinguishing features between these mechanisms [8], [10], [11]. Side-chain rotamers distributions and\or redistributions observed in the conformational transitions are pivotal mechanistic features of protein functions. Side-chain rotamer libraries have been widely used in theoretical conformational and X-ray crystallographic models.

Recently site-directed tryptophan fluorescence (SDTF) was used to assign rotameric distributions in the alpha helix of tear lipocalin and to detect conformational changes involving side-chain rearrangement [13]. Recent work [14]–[21] forms the basis for (SDTF) as an effective tool to study the relationship between protein structure, dynamics and function. The rotameric distribution model derived from SDTF (RD-SDTF) uses a three-site jump rotamer model of χ_1_ (180° (*t*); −60° (*g* ^−^); +60° (*g* ^+^)) to assign Trp fluorescence lifetimes [13], [22]. Rotamer libraries derived from the extensive X-ray crystallographic data support a model with three canonical rotamers for χ_1_ angles [23]–[25]. Non-canonical rotamers (χ_1_ angles) comprise less than 1% of the rotamer library.

Extensive experimental and theoretical research on conformationally restricted peptides in which Trp side-chain may assume certain rotamers (assigned by NMR spectroscopy) have assigned fluorescence lifetimes to particular rotamers [17], [26], [27]. Because the Trp side-chain in each rotamer is uniquely positioned with respect to the carbonyl group, distinct fluorescence lifetimes have been convincingly assigned to a three-site rotamer model. Shorter than expected fluorescence lifetimes can be explained by the fact that the side-chains of some amino acids, such as, Lys, Tyr, Gln, Asn, Glu, Asp, Cys, and His, may quench Trp fluorescence with various efficiencies [14]. The side-chains of Lys and Tyr residues may quench Trp fluorescence by an excited-state proton transfer mechanism [14], [28]–[30]. The side-chains of Gln, Asn, Glu, Cys and His quench Trp fluorescence by excited-state electron transfer [14]. Electron transfer from the excited indole ring to the nearest C-atom of the carbonyl group is the principal mechanism of fluorescence quenching [14], [19], [20], [31].

Experimental evidence supports a rotamer switch mechanism for ligand binding in lipocalins [6], [32]–[34]. A protonation\deprotonation of Glu27 regulates the loop AB movement that switch the rotameric states of side-chains of several amino acids and affect ligand binding [6], [34], [35]. The lipocalin family members have a limited conserved homology (∼20%) in amino acid sequence despite sharing the ligand binding barrel comprised from eight antiparallel β-strands with a repeated +1 topology [36]. The variation in primary structure in lipocalins seems to control the variation in individual ligand binding functions. In TL, a capacious ligand binding scaffold confers promiscuity in ligand binding [6], [37]. In human tears, TL binds a wide variety endogenous ligands, such as fatty acids, alkyl alcohols, glycolipids, phospholipids, cholesterol and etc. [38], [39]. TL also binds various exogenous ligands [6], [37], [40]–[42]. In TL, several amino acids are critical for its functions. For example, Trp17, is highly conserved in analogous regions within the lipocalin family and is essential for the structure and function. A single lifetime population (97%) befits a single rotamer that accounts for the fluorescence decay of Trp17 [43]. The long-range cation-π interaction evident between Trp17 and Arg18 is feasible for one particular rotameric state of Trp17 [34]. Therefore, Trp17 resides in a restricted environment and samples conformations within a single rotamer energy well.

Backbone conformations as well as side-chain interactions determine rotamer distributions of amino acids in proteins [44]. In this study long-range side-chain interactions were found to influence rotamer distributions of the side-chain located at position 130 (Trp130) of TL. Trp130 located in the main α-helix of TL interacts with distant residues Val113 and Leu115 of the β-strand H. The C^α^ atom of the Trp130 lies 7.5 and 6.0 Å distant to the C^α^ atoms of the Val113 and Leu115, respectively [45]. These sites were mutated to various combinations of Gly, Ala and/or Phe residues to modify side-chain interactions. Results are discussed in terms of structural and rotamer population changes.

Quantitative description of less populated side-chain rotamers are challenging in structural biology, particularly, in X-ray crystallography. RD-SDTF may contribute significantly to resolve low frequency populations of side-chain rotamers. A rotamer residing at a higher energy well is important for characterization of functionally active low-populated (aka, invisible) excited protein states.

## Materials and Methods

### Materials

Ficoll PM 70, Sucrose and all materials used in preparation of the dilute solutions of the mutant proteins were purchased from Sigma-Aldrich (St. Louis, MO).

### Site-directed mutagenesis and plasmid construction

TL cDNA [46], previously synthesized in PCR II (Invitrogen), was used as the template to clone the TL gene spanning bases 115–592 of the sequence [47] into pET 20b (Novagen, Madison, WI). To construct the native protein sequence as found in tears, flanking restriction sites were added to Ndel and BamHI. However, the initiating methionine was not removed [48].

The TL mutant W17Y\F130W, which was previously characterized [13], [37], was used as a template to construct the mutants to modify the long-range interacting residues. Mutants were constructed with oligonucleotides (Invitrogen) using QuikChange II site-directed mutagenesis kit (Stratagene) and obtained cDNA with introduced point mutation was confirmed by sequencing. Amino acid 1 corresponds to His, bases 115–118, according to previously published work [47].

To test the influence of long-range side chain interactions on rotamer distributions of Trp residues, Trp130 of TL, was selected. Trp 130 is deeply buried in the hydrophobic interface located between the α-helix and β-barrel. On the basis of prior work [37], [45] Val113 and Leu115 are distant residues that interact with Trp 130 and were mutated to probe the potential effect on rotamers as follows: W17Y\F130W (for simplicity W130), W17Y\F130W\V113G (W130G113), W17Y\F130W\V113A (W130A113), W17Y\F130W\V113F (W130F113), W17Y\F130W\L115G (W130G115), W17Y\F130W\L115A (W130A115), W17Y\F130W\L115F (W130F115), W17Y\F130W\V113G\L115A (W130G113A115), W17Y\F130W\V113A\L115A (W130A113A115) and W17Y\F130W\V113F\L115F (W130F113F115).

### Expression and purification of mutant proteins

The mutant plasmids were transformed in E. Coli, BL 21 (DE3), cells were cultured and proteins were expressed, purified, and analyzed as described [37], [49]. The expressed mutant proteins were used without additional enrichment with ligand. Previously, it has been shown that mutant proteins expressed in E. Coli as well as the native protein contain various fatty acid ligands including palmitic acid [38], [40]. Concentrations of the mutant proteins were determined using the molar extinction coefficient of TL (ε_280_ = 13760 M^−1^cm^−1^) [50]. For the mutants containing Trp130, the molar extinction coefficients were calculated to be 15040 M^−1^cm^−1^.

### Absorption Spectroscopy

UV absorption spectra of the mutants of TL were recorded at room temperature using a Shimadzu UV-2400PC spectrophotometer. All experiments were performed in 10 mM sodium phosphate, pH 7.3. To increase the accuracy of fluorescent quantum yield values of Trp mutants, the spectra were corrected for light scattering as described elsewhere [49].

### CD spectral measurements

Far-UV CD spectra were recorded for all mutants at room temperature on a Jasco J-810 spectropolarimeter. The path length was 0.2 mm. The concentrations of the proteins were about 0.9 mg/ml. All CD experiments were performed in 10 mM sodium phosphate, pH 7.3. TL does not form any dimers at this concentration (0.9 mg/ml) and even much higher [51]. Nine scans of each CD spectrum were averaged to improve signal/noise ratio.

The CD spectra of the mutants were analyzed to calculate the content of secondary structure using a CDPro software that has three algorithms: CONTINLL, CDSSTR and SELCON3 [52]. In calculations, the protein reference set was SMP56, which comprised of 43 soluble- and 13 membrane protein references. The normalized root-mean-square deviation (nrmsd) values between the experimental and calculated spectra were used to judge the goodness of fit. The nrmsd value was calculated as:
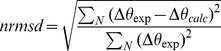



Where 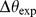
and 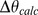
are the experimental and calculated mean residue ellipticity, respectively. N is the number of data points. In estimations of secondary structures of mutants, all above mentioned algorithms yield similar data. However, CONTINLL generated consistently better fits. Therefore, data for secondary structure estimates are given for CONTINLL only. It should be noted that CONTINLL in contrast to CONTIN implements variable selection by removing the least similar proteins. In calculations we have selected the basis which includes most proteins in the reference set. The calculations with SMP56 and SP43 (only 43 soluble proteins included to SMP56) revealed very similar results. Furthermore, evaluation of CD spectra of soluble proteins with SMP56 yields higher accuracy compared to that obtained with SP43 [53].

### Steady-State Fluorescence spectroscopy

Steady-state fluorescence measurements were made with a JobinYvon-SPEX (Edison, NJ) Fluorolog tau-3 spectrofluorometer. The bandwidths for excitation and emission were 2 nm and 3 nm, respectively. The excitation λ of 295 nm was used to ensure that light was absorbed almost entirely by a tryptophanyl group. Protein solutions with about 0.07 OD at 295 nm were analyzed. All spectra were obtained from samples in 10 mM sodium phosphate at pH 7.3. The fluorescence spectra were corrected for light scattering from buffer and then for the instrument response function by means of the appropriate correction curve. The quantum yields of the Trp residues in the proteins were calculated using a fluorescence standard, NATA (N-acetyl-L-tryptophanamide). The quantum yield of NATA was taken as 0.13 [54]. To improve accuracy in calculations of the quantum yields, the blue sides of the emission spectra were constructed using the log-normal function as described previously [49].

### Time-resolved fluorescence measurement

Time-resolved intensity decay data were obtained using a HORIBA JobinYvon MF^2^ phase/modulation multi-frequency domain fluorometer. The excitation wavelength was 295 nm (LED). Emission was detected through a monochromator at the fluorescence λ_max_ of each mutant. P-terphenyl in ethanol was used as a reference standard (τ = 1.05 ns). For some mutants, fluorescence lifetime measurements were performed in the buffer containing 30% (v/v) of sucrose or 25 % (v/v) of Ficoll 70. Data analyses were performed with a nonlinear least-squares program from the Center for Fluorescence Spectroscopy (M. L. Johnson), University of Maryland at Baltimore, School of Medicine (Baltimore, MD). The goodness of fit was assessed by the χ^2^ criterion.

The intensity decay data were analyzed in terms of the multi-exponential decay law:
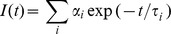
where 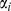 and 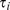 are the normalized pre-exponential factors and decay times, respectively. The fractional fluorescence intensity of each component is defined as 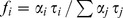.

Intensity- (mean lifetime) and amplitude averaged (corresponding to quantum yield) lifetimes were calculated as 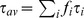 and 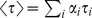, respectively.

## Results and Discussion

### Circular Dichroism

**Figure 1:**
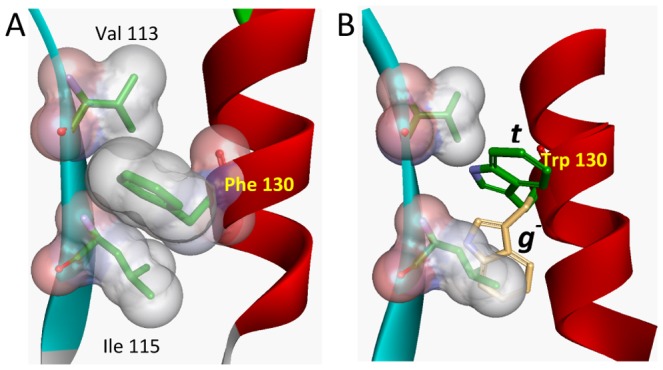
Relative orientations of the side-chains at position 130 and its long-range interaction partners in β-barrel-α-helix interface of TL. A. Relative orientation of native Phe130 (χ_1_ = 180°). B. The rotamers *t* (χ_1_ = 180°) and *g* ^−^ (χ_1_ = −60°) of Trp at position 130. The mutation to introduce Trp at position 130 and rotamer assignment were generated from the PDB file (1XKI) with DS Visualizer 3.0 (Accelrys Inc.).

**Figure 2:**
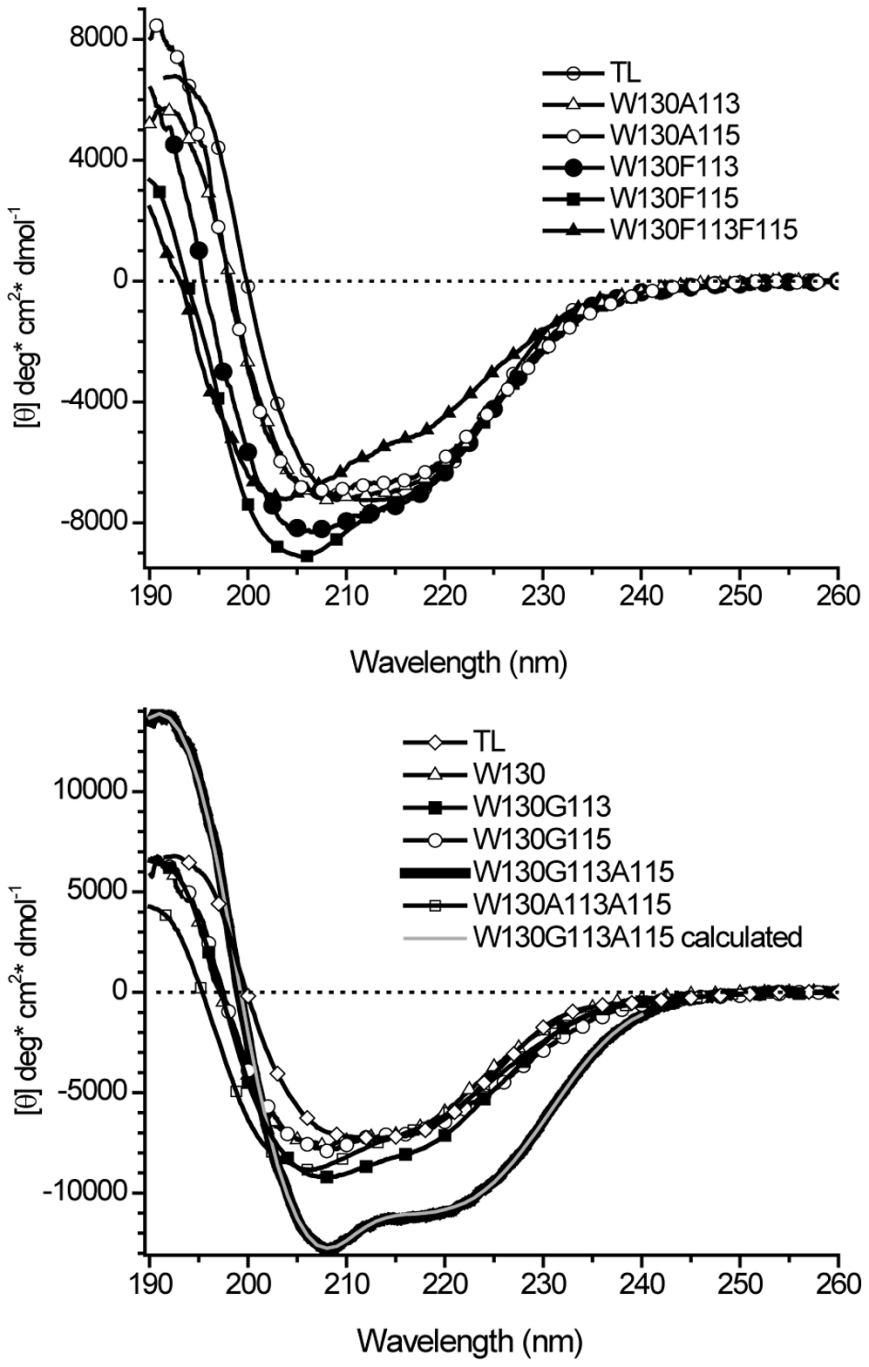
Far-UV CD spectra of the mutants with single Trp130 in which long-range interaction (the positions 113 and 115) were modified. The CD spectrum of native TL is shown for comparison. For clarity the spectra are divided in two sets (A) and (B).

The side-chain of Trp130 located in the main α-helix interacts with the side-chains of Val113 and Leu115 of the β-strand H (Fig. 1) [13], [37]. In lipocalins, α-helix- β-barrel (mostly via the β-strand H) interactions stabilize β-sheet stability as monitored in unfolding\refolding kinetics [55], [56]. Therefore, mutations that modify α-helix- β-barrel interactions may induce structural changes. Val133 and Leu115 were substituted with Gly, Ala and Phe residues at 113 and/or 115 (see Materials and Methods). To monitor structural changes CD spectroscopy was applied to these mutants. CD spectra of all mutants are shown in Figure 2. Changes observed in the CD spectrum of W130 compared to that of native TL (Fig. 2B) have been attributed to alteration in packing of secondary structural elements generated by the introduction of a side-chain bulkier than Phe130 (Fig. 1B) [37]. The crystal structure of TL corroborates this notion [45]. The side chain of the Phe130 makes contact with two side chains of the residues, Val113 and Leu115 (Fig. 1) [45]. Substitutions of Val113 and Leu115 to Gly or Ala in the mutants W130A113, W130A115, W130G115, W130G113 and W130A113A115 show minimal distortion. However, much bigger alterations were observed for substitutions of these sites to bulkier side-chains (mutants, W130F113, W130F115 and W130F113F115). The biggest change is observed in the mutant W130G113A115 (Fig. 2). The large increase in CD intensity and formation of two new negative peaks at about 208 nm and 222 nm indicate a significant increase in α-helix content. The calculated values for secondary structure content of the mutants are shown in Table 1. Consistent with differences observed in CD spectra, the mutant W130G113A115 shows the biggest changes in secondary structure, β-sheet to α-helix transition. In this mutant, β-structure decreased 18.2% (from 34.8% to 16.6%) and concomitantly, α-helix increased 22.2% (from 10.4% to 32.6%). The β-structure to α-helix transitions, are also observed but to a smaller degree in the mutantsW130G113, W130G115 and W130A113A115 (Fig. 2 and Table 1). Taking into account that CDPro estimates α-helix content in a protein by ∼5% accuracy [57], one may conclude that these mutants show tendencies for the β-structure to α-helix transitions. Two of them correspond to the mutants in which interactions with Trp130 were removed for one (position 113 or 115) of the two sites. In the third mutant, W130A113A115, both sites were replaced to insert the smallest hydrophobic group as a side-chain. In addition to its small size, Ala does not exhibit χ_1_ rotamers. Perhaps, this mutant reflects the situation where possible interactions with Trp130 are minimal for side-chains. In the mutant W130A113A115, the removal of one of these interaction sites, namely Ala113 (mutant W130G113A115), leads to dramatic increase in the β-structure to α-helical transition (Figures 1 and 2, Table 1). These findings indicate that TL has very high propensity for α-helix formation. Another lipocalin, β-lactoglobulin, showed transient α-helix formation in a folding intermediate state [55], [56]. The results indicate the importance of long-range interactions in determining the native folds of the proteins. A Monte Carlo simulation of protein folding to show the relative importance of short- (residues separated by up to three amino residues along the polypeptide chain) and long-range (residues, separated by four or more residues along the polypeptide chain, but in close proximity) interactions is revealing [58]. Both short- and long-range interactions are necessary to achieve a native fold of protein. Short-range interactions, which are dominant in establishing the local structure, alone are not satisfactory to achieve and stabilize a native fold. This is fundamental to the model of hierarchic protein folding [1]–[3] Consistent with this view, mutants that vitiate interactions between the main α-helix and β-sheet produce stable non-native α-helical formation. The changes are associated with the lack of long range tertiary interactions. It seems reasonable that the transient α-helix formation in β-lactoglobulin occurs only transiently prior to establishing the more long range interactions [55], [56].

**Table 1:** Secondary structure content for TL mutants at pH value of 7.3 determined from CD spectra.

Protein	Program	α- helix (%)^a^			β- strand (%)^b^			turn (%)	unrd^c^ (%)	nrmsd
		H(r)	H(d)	∑H	S(r)	S(d)	∑S			
TL	CONTINLL	4.5	6.9	**11.4**	23.1	12.2	**35.3**	23.2	30.1	0.029
W130	CONTINLL	3.7	7.0	**10.7**	22.5	12.3	**34.8**	23.0	31.4	0.028
W130G113	CONTINLL	7.2	8.8	**16.0**	19.0	11.1	**30.1**	21.8	32.0	0.014
W130A113	CONTINLL	4.6	7.1	**11.7**	22.3	11.8	**34.1**	22.7	31.5	0.022
W130F113	CONTINLL	3.4	6.3	**9.7**	21.9	12.4	**34.3**	22.0	34.1	0.021
W130G115	CONTINLL	7.1	8.9	**16.0**	19.3	11.2	**30.5**	21.7	31.9	0.019
W130A115	CONTINLL	4.6	7.3	**11.9**	22.4	12.0	**34.4**	23.0	30.7	0.023
W130F115	CONTINLL	4.2	7.7	**11.9**	19.3	11.5	**30.8**	23.1	34.2	0.013
W130G113A115	CONTINLL	16.7	15.9	**32.6**	8.9	7.7	**16.6**	22.1	29.7	0.012
W130A113A115	CONTINLL	5.6	8.4	**14.0**	18.3	11.4	**29.7**	23.0	33.3	0.010
W130F113F115	CONTINLL	1.9	5.8	**7.7**	23.1	12.4	**35.1**	22.9	33.8	0.024

a H(r) and H(d) are for regular and distorted α-helix, respectively. ∑H = H(r)+H(d).

b S(r) and S(d) are for regular and distorted β-strand, respectively. ∑S = S(r)+S(d).

c unrd is for unordered fraction.

Thus, in folding of the lipocalins the intermediate states that are rich in α-helix content refer to a situation in which the main α-helix did not establish long-range tertiary interactions.

### Steady-State Fluorescence Spectroscopy

**Figure 3:**
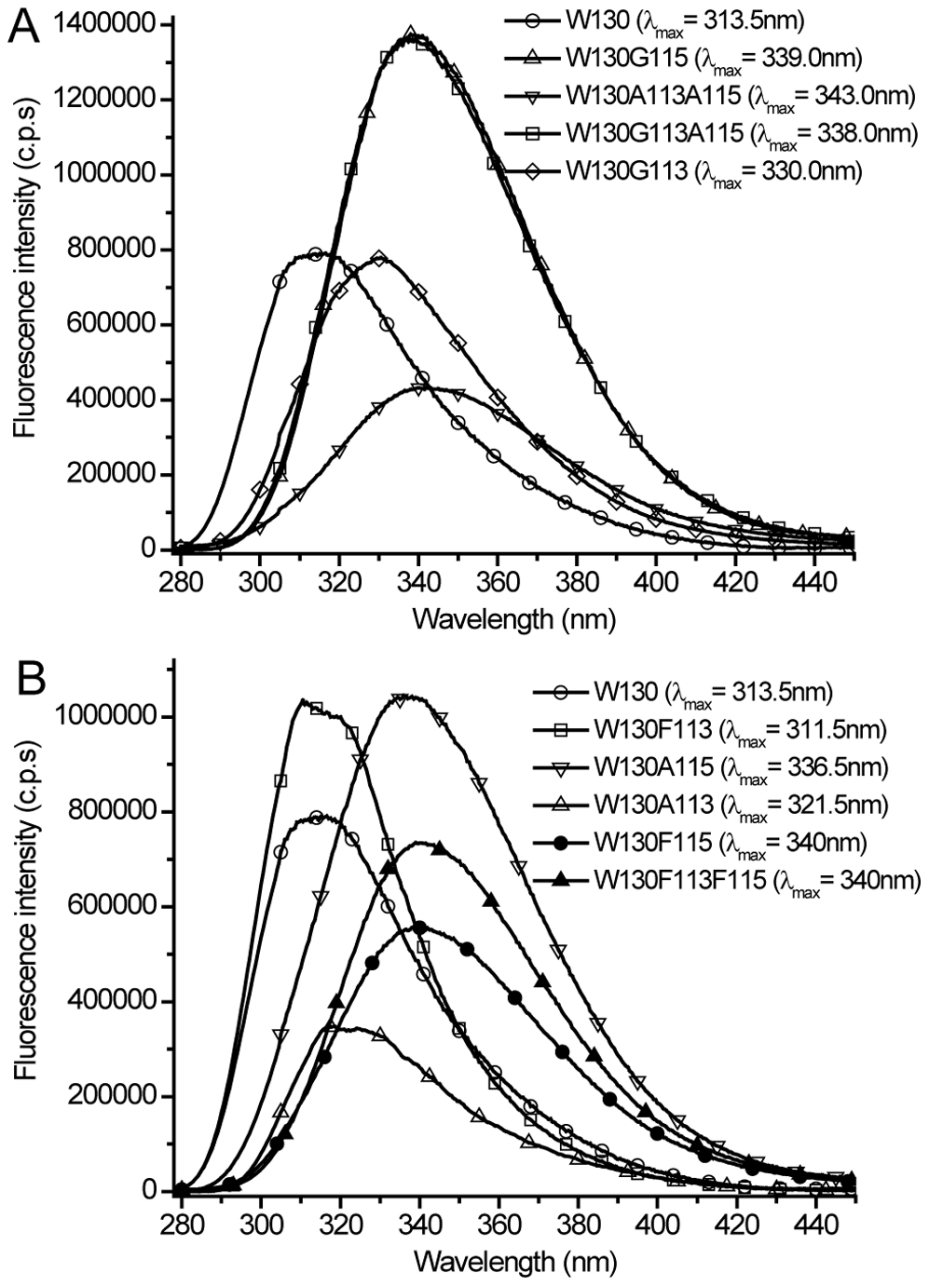
The corrected fluorescence spectra of the mutants with single Trp130 in which long-range interaction (the positions 113 and 115) were modified. Fluorescence intensities were normalized to the same absorbance (0.05) at 295 nm to reflect respective quantum yield values. For clarity the spectra are divided in two sets (A) and (B).

The fluorescence spectra of the mutants are shown in Figure 3. Trp130 in the native environment has a fluorescence λ_max_ at about 313.5 nm. Substitution of Val113 with Phe shifts λ_max_ even more blue side to 311.5 nm. Vibrational structures in the spectrum are more pronounced. However, Phe residue introduced at position 115 significantly shifts the fluorescence spectrum to the red side, to 340 nm (Fig. 3). As can be seen from Figure 1B, the side-chain at position 115 creates more steric restriction for Trp130 than position 113. The distance between the C^α^ atoms of Trp130 and Leu115 is 6.0Å in contrast to 7.5Å that of between Trp130 and Val113 (Fig. 1). Accordingly, somewhat higher distortion of the secondary structure is noted in CD data for W130F115 compared to that of W130F113 (Fig. 2 and Table 1). It should be noted that while the fluorescence spectrum reports the properties of the immediate environment of the chromophore, a CD spectrum reflect a global secondary structure of the protein. Therefore, depending on the location of perturbation, small changes observed on global scale could be dramatic on a local scale. Interestingly, in the mutant W130G115 where interaction between the side-chain of Trp130 and that at position 115 is removed, the fluorescence λ_max_ is red shifted significantly to 339 nm. CD spectrum of the mutant W130G115 is identical to that of W130. Restoration of the interaction occurs minimally in the mutant W130A115 and shifts the fluorescence λ_max_ to the blue side from 339.0 nm to 336.5 nm. The native Leu115 appears to create a hydrophobic environment for the side-chain of Trp130. Removal of the side-chain at position 113 (Fig. 1) in the mutant W130G113 shifts fluorescence λ_max_ to red side from 315.5 nm to 330 nm. In contrast W130G115 shifts fluorescence λ_max_ to 339 nm. Restoration of the interaction occurs minimally with a methyl group in the mutant W130A113 and significantly blue shifts fluorescence λ_max_ from 330 nm to 321.5 nm compared to 336.5 nm observed in W130A115. The results indicate that the side-chain in the position 113 also influence the environment of Trp130. However, the alteration of the local structure is minimal. Hence, the red shift noted for the spectra of Trp130 (from 24 to 29 nm) in some mutants indicates greater exposure to solvent. This is further evidence that long range interactions were eliminated by the mutations that do not produce distortion in the local structure and feature two lifetimes in fluorescence decays.

### Time-Resolved Fluorescence and Rotamer Distribution

**Figure 4:**
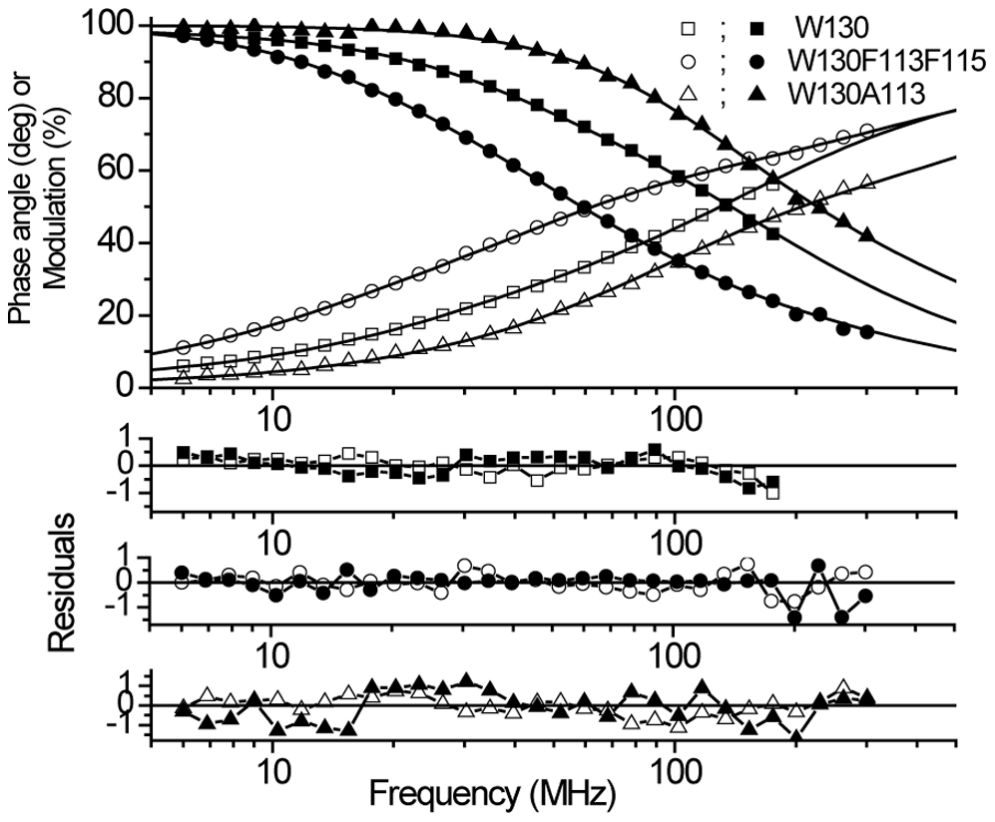
Representative phase angle (open symbols) and modulation (solid symbols) fluorescence lifetime data of TL mutant W130, W130F113F115 and W130A113 at pH 7.3. Solid lines represent the best bi- or tri-exponential fit for the parameters given in Table 1.

CD and steady-state fluorescence data reported above reveal the significance of the long-range interactions (with Trp130) for the local structural and environmental characteristics. The mutations at positions 113 and 115 with Gly, Ala and Phe create wide variety of situations that are discussed above. To reveal how modification of the long-range interaction influences the rotamer population of Trp 130, time-resolved measurements were performed for all mutants considered in this study. Figure 4 shows representative fluorescence intensity decay curves and best fits for mutants W130, W130F113F115 and W130A113. Fluorescence lifetime parameters are shown in Table 2. To ensure that lifetimes of Trp 130 are not influenced by solvent relaxation, fluorescence decay curves were measured with 30% sucrose and 25% Ficoll 70. At these concentrations, they have same viscosity, but osmolarity values greatly differ from each other due to differences in molecular mass [59]. One would expect that solvent relaxation around excited Trp residues should be more diminished in 30% sucrose. As can be seen from Table 2 the fluorescence lifetimes as well as pre-exponential parameters are not influenced much by solvent viscosity. Therefore, solvent relaxation processes minimally impact the fluorescence lifetime parameters.

**Table 2:** Fluorescence lifetime parameters for the mutants with single Trp130 located in the α-helix of human tear lipocalin in various long-range interacting partners located in the positions 113 and 115.

Mutant	aα_1_	α_2_		bτ_1_ (ns)	τ_2_ (ns)		c<τ>(ns)	dτ_av_ (ns)	eQ	fκ_r_ (s^−1^)	χ^2^
W130	0.84 (0.01)	0.16 (0.01)		1.17 (0.02)	4.20 (0.12)		1.65 (0.05)	2.40 (0.14)	0.07	42·10^6^	0.7
W130 Sucrose	0.80 (0.01)	0.20 (0.01)		1.13 (0.02)	4.15 (0.11)		1.73 (0.05)	2.58 (0.14)			0.8
W130 Ficoll	0.77 (0.01)	0.23 (0.01)		0.93 (0.03)	3.50 (0.10)		1.52 (0.05)	2.29 (0.13)			0.9
Global fitting; Global parameter- α_i_											
W130	0.77 (0.01)	0.23 (0.01)		1.17 (0.02)	4.20 (0.14)		1.87 (0.06)	2.74 (0.16)			0.8
W130 Sucrose				1.13 (0.02)	4.14 (0.12)		1.82 (0.05)	2.70 (0.15)			
W130 Ficoll				0.93 (0.02)	3.50 (0.09)		1.52 (0.04)	2.29 (0.12)			
W130F113	0.81 (0.02)	0.19 (0.01)		1.16 (0.03)	3.59 (0.14)		1.62 (0.06)	2.18 (0.13)	0.08	49·10^6^	0.8
W130F113 Sucrose	0.79 (0.02)	0.21 (0.02)		1.00 (0.03)	3.13 (0.12)		1.45 (0.05)	1.97 (0.12)			0.9
W130F113 Ficoll	0.78 (0.01)	0.22 (0.01)		0.83 (0.02)	3.12 (0.10)		1.33 (0.04)	2.01 (0.12)			1.0
Global fitting; Global parameter- α_i_											
W130F113	0.78 (0.01)	0.22 (0.01)		1.16 (0.04)	3.61 (0.16)		1.70 (0.06)	2.31 (0.15)			0.9
W130F113 Sucrose				1.00 (0.03)	3.12 (0.13)		1.47 (0.05)	1.99 (0.12)			
W130F113 Ficoll				0.83 (0.03)	3.10 (0.10)		1.33 (0.05)	1.99 (0.12)			
W130F115	0.42 (0.02)	0.42 (0.01)	0.16 (0.02)	0.58 (0.05)	2.47 (0.18)	6.12 (0.28)	2.26 (0.16)	3.85 (0.46)	0.07	31·10^6^	0.8
W130F113F115	0.43 (0.02)	0.43 (0.02)	0.14 (0.02)	0.65 (0.04)	3.19 (0.15)	8.67 (0.64)	2.87 (0.24)	5.26 (0.80)	0.09	31·10^6^	0.9
W130A115	0.57 (0.01)	0.43 (0.01)		1.10 (0.03)	4.64 (0.06)		2.62 (0.06)	3.79 (0.13)	0.11	42·10^6^	0.7
W130A115 Sucrose	0.60 (0.01)	0.40 (0.01)		1.05 (0.03)	4.66 (0.07)		2.49 (0.06)	3.75 (0.14)			0.9
W130A115 Ficoll	0.63 (0.01)	0.37 (0.01)		0.89 (0.03)	4.42 (0.07)		2.20 (0.06)	3.52 (0.14)			1.0
W130A113	0.51 (0.01)	0.49 (0.01)		0.32 (0.04)	1.44 (0.03)		0.87 (0.03)	1.23 (0.06)	0.04	46·10^6^	1.3
W130A113A115	0.44 (0.02)	0.40 (0.01)	0.16 (0.03)	0.56 (0.05)	2.51 (0.22)	5.89 (0.33)	2.18 (0.22)	3.71 (0.63)	0.06	28·10^6^	0.8
W130G113	0.46 (0.02)	0.54 (0.02)		0.94 (0.05)	3.32 (0.06)		2.23 (0.08)	2.86 (0.15)	0.09	40·10^6^	1.1
W130G115	0.48 (0.01)	0.52 (0.01)		1.21 (0.05)	5.48 (0.08)		3.43 (0.07)	4.76 (0.16)	0.13	38·10^6^	1.0
W130G113A115	0.47 (0.01)	0.53 (0.01)		1.24 (0.05)	4.91 (0.08)		3.19 (0.07)	4.24 (0.15)	0.13	41·10^6^	0.9

a Normalized pre-exponential factor.

b Decay time.

c Amplitude-averaged lifetime.

d Intensity-averaged lifetime.

e Quantum yield.

f Radiative rate constant.

Numbers in parentheses indicate standard deviations. Sucrose- 30% v/v. Ficoll- Ficoll PM 70, 25% v/v.

Most mutants show two lifetimes. Considering the three-site jump rotamer model for χ_1_ it seems valid to assign Trp130 to an α-helix conformation with *t* and *g* ^−^ rotamers. The *g* ^+^ rotamer for a Trp residue is prevented by steric restriction with the *i*-3 backbone atom. However, the mutants W130F115, W130F113F115 and W130A113A115 display three lifetimes. Two mutant proteins (W130F115 and W130F113F115) show significant alterations in secondary structures (Fig. 2). In the mutant W130A113A115, structural changes are greater than in the base mutant (W130). Because three lifetimes are not possible for Trp fluorescence located in a typical α-helix, these three mutants have distortions at the main α-helix in the vicinity of position 130. The most likely explanation is that the distortions in the mutants occur only in sub-population of the proteins, therefore, increasing structural heterogeneity of the protein. Previously, the lifetimes of Trp 130, 1.17 ns and 4.20 ns, were assigned to *t* and *g* ^−^ rotamers, respectively. This was based on the structural consideration of TL (Fig. 1), rotamer distribution data for TL [13] and model peptides [60]. In model α-helical peptides, the long lifetime component (*g* ^−^) was about 7 ns. On this basis, for the mutants, W130F115, W130F113F115 and W130A113A115, the shortest lifetime components (from 0.55 ns to 0.65 ns) should be assigned to *g* ^+^ component. These three mutants exhibit the third rotamer (*g* ^+^) conformation in about 43% of the population. However, an alternative assignment for three lifetimes is attractive. Shorter lifetimes 0.56 ns and 2.51 ns (W130A113A115), which are within the range observed for mutants with two lifetimes, could be interpreted as *t* and *g* ^−^ rotamers, respectively. That leaves the 5.89 ns component to the rotamer g^+^ with 16% population. Such an interpretation would suggest a much smaller scale distortion. This interpretation also has a solid foundation. In certain peptides the lifetime of the *g* ^+^ rotamers of Trp can be about 6 ns [19]. Further investigation is needed for unambiguous assignments of three lifetimes. However, the basic fact remains the same. Occurrence of three fluorescence lifetimes for Trp located in α-helix means that the local α-helix conformation is distorted. Inspection of rotamer distributions within the other mutants is also revealing. Only in two mutants, W130 and W130F113, in which the side-chain of Trp130 is extremely buried with hydrophobic groups (fluorescence λ_max_ are 313.5 and 311.5 nm, respectively), show a population with a predominant *t*-rotamer (Fig. 3 and Table 2). Figure 1B demonstrates that the *t*-rotamer would be less sterically constrained than the g^−^ rotamer in these conditions and expected to be dominant. Mutants W130G113, W130G115, W130A113 and W130A115 result in the elimination of a dominant single rotamer and coincide with vitiation of the long-range interaction sites for Trp130. Mutant W130G113A115 shows a significant increase in α-helix content and a rotamer distribution in two states neither of which is dominant. Thus, for Trp located in α-helix, the dominant single rotamer is expected in situations where interaction partners have bulky side-chains that do not distort local structure.

## Conclusion

Specific long-range protein interactions critically affect the folding and conformation state of proteins. The current study shows that these interactions are effectively probed with site-directed tryptophan fluorescence in combination with rotameric modeling (RD-SDTF). Trp fluorescence lifetimes were assigned to tree-site rotamer model of χ_1_. RT-SDTF reveals features that govern the rotamer populations of Trp residues. Mutations of the long-range interaction partners (Val113 and Leu115 of β-strand H) of Trp130 (introduced to the α-helix) create following three distinct situations for the tryptophan: 1. Trp in α-helix interacts with distant residues. Consequently, this situation describes an α-helix in which both short- and long-range interactions are well established. 2. The local α-helical conformation of Trp is distorted by mutations. 3. Simultaneous substitutions of long-range interaction sites to Gly and\or Ala eliminate specific interactions of Trp in the α-helix with distant residues, leaving only short-range interactions. In RT-SDTF fluorescence lifetime distributions reveal the hierarchical nature of rotamer populations. The short range interactions extant in the backbone conformation of the secondary structure restrict rotamer populations of Trp residue. Long-range interactions in a native fold further tune the distribution of rotamer populations according to the nature of the interacting partners. RT-SDTF and CD measurements in TL indicate that the removal of long-range interaction leads to formation of a non-native α-helix. This situation parallels the conditions in folding of proteins where the secondary structural elements do not establish long-range tertiary interactions.
